# Mental Health and COVID-19 in University Students: Findings from a Qualitative, Comparative Study in Italy and the UK

**DOI:** 10.3390/ijerph20054071

**Published:** 2023-02-24

**Authors:** Ilaria Riboldi, Chiara Alessandra Capogrosso, Susanna Piacenti, Angela Calabrese, Susanna Lucini Paioni, Francesco Bartoli, Cristina Crocamo, Giuseppe Carrà, Jo Armes, Cath Taylor

**Affiliations:** 1 Department of Medicine and Surgery, University of Milano-Bicocca, Via Cadore 48, 20900 Monza, Italy; 2 Division of Psychiatry, University College London, Maple House 149, London W1T 7BN, UK; 3 Faculty of Health and Medical Sciences, School of Health and Sciences, University of Surrey, Stag Hill, Guildford GU2 7XH, UK

**Keywords:** COVID-19, interviews, mental health, university students

## Abstract

Introduction: COVID-19 restrictions introduced several changes in university academic and social experience. Self-isolation and online teaching have amplified students’ mental health vulnerability. Thus, we aimed to explore feelings and perspectives about the impact of the pandemic on mental health, comparing students from Italy and the UK. Methods: Data were collected from the qualitative portion of “the CAMPUS study”, longitudinally assessing mental health of students at the University of Milano-Bicocca (Italy) and the University of Surrey (UK). We conducted in-depth interviews and thematically analysed the transcripts. Results: The explanatory model was developed from four themes identified across 33 interviews: anxiety exacerbated by COVID-19; putative mechanisms leading to poor mental health; the most vulnerable subgroups; and coping strategies. Generalised and social anxiety resulted from COVID-19 restrictions by being associated with loneliness, excessive time online, unhealthy management of time and space and poor communication with the university. Freshers, international students, and people on the extremes of the introversion/extroversion spectrum, were identified as vulnerable, while effective coping strategies included taking advantage of free time, connection with family and mental health support. The impact of COVID-19 was mostly related to academic issues by students from Italy, whereas to the drastic loss of social connectedness by the UK sample. Conclusions: Mental health support for students has an essential role, and measures that encourage communication and social connectedness are likely to be beneficial.

## 1. Introduction

Higher education settings can expose students to psychological and mental health distress, especially during the first year of their academic course [1,2]. Indeed, the transition to university is often characterized by an increase in academic pressure, as well as major changes in lifestyle habits [3]. Common mental health disorders have risen significantly among university students in terms of both incidence and severity over the past decade, with anxiety and depressive symptoms being the most represented conditions [4,5,6,7]. Moreover, the worldwide spread of the COVID-19 pandemic has affected all major sectors, including higher education, thus intensifying performance and social challenges faced by students [8,9,10]. According to national policies, several restriction measures were widely implemented at multiple points over the course of the pandemic [11]. Indeed, in the UK, during the lockdowns, students from faraway countries had to remain on campus [12,13], while in Italy, the stay-at-home instruction led to a huge migration to nearby hometown residences [14,15,16]. In addition, following policy guidelines, the whole university system in both countries shifted to online teaching [17]. Self-isolation and the negative impact of the compulsory online learning amplified students’ psychological burden, leading to an increased level of anxiety, depression, and stress [18,19,20,21]. Moreover, COVID-19 restrictions influenced students’ motivation, with a negative impact on academic performance, and fed their sense of uncertainty about the future [22,23,24], also favouring an unhealthy management of individual time and space [16,25,26]. All of these conditions, together with poor communication between students and the institution, contributed to the exacerbation of mental health distress [27,28,29]. During the pandemic, first-year students, as well as international ones were among the most vulnerable categories [1,4,30]. Indeed, university social connectedness, weakened during the pandemic, is significant in favouring the adjustment to an unfamiliar environment [31,32]. Therefore, several educational institutions have prioritized preventative approaches, through the enhancement of existing well-being centres and psychoeducation programmes [33,34], along with the implementation of specific digital interventions [35,36,37].

Despite students’ perspectives on the topic have been previously investigated through a qualitative approach [38,39], possible failures in the national educational system, as well as specific accommodation and lifestyle habits could have represented additional triggers in the onset and the worsening of mental health symptoms during the pandemic, both in Italy and the UK [16,40,41]. Thus, we carried out in-depth interviews with the aim to explore students’ experiences, feelings, and perspectives about the impact of COVID-19 on mental health in university settings and to compare key data between respondents from Italy and the UK.

## 2. Materials and Methods

### 2.1. Study Design and Setting

This study was reported in line with the COnsolidated criteria for REporting Qualitative research (COREQ) checklist [42]. Data were derived from the qualitative portion of the “Caring and Assessing Mental health of student Populations at Unimib and uniSurrey: the CAMPUS study”, a large ongoing project longitudinally assessing the mental health of university students enrolled at the University of Milano-Bicocca (Unimib, Milano, Italy) and the University of Surrey (UoS, Guildford, UK). In-depth interviews were used to explore students’ experiences, feelings, and perspectives on the relationship between mental health and the COVID-19 pandemic. We selected semi-structured in-depth interviews to allow participants to freely express themselves, facilitating a conversational focus on specific aspects of mental health and enabling comparisons across participants. According to our approach to in-depth interviews, we relied on established research supporting the validity of a number of about 10–20 interviews in order to reach a likely sufficient data saturation [43,44]. The existing literature on student mental health informed the interview guide development (Supplementary File S1). For the purpose of this paper, we considered the interview section focusing on the COVID-19-related issues. The interview framework was piloted with three students to ensure understandability and face validity in relation to the topics covered, as well as minimal distress and maximum relevance. A university steering committee was established to provide advice and promote the project. Interviews were scheduled via Microsoft Teams online platform by a researcher (I.R.) between September 2021 and April 2022. During this significant interval of time, COVID-19 restrictions underwent transformations both in Italy and the UK, following the main epidemiological waves across variously strict measures. Interviews were audio-recorded and transcribed verbatim (A.C., C.A.C., S.L.P., S.P.) and lasted 32 min on average.

### 2.2. Participants

Participants were eligible to participate if they were 18 years of age or older and currently enrolled at one of the two universities. They were purposively sampled according to gender and age distributions, nationality, programme of study, and living accommodation to provide a variety of perspectives and experiences. Students meeting the inclusion criteria were approached by key site contacts via email and invited to participate in the study. Email invitations were repeated at reasonable intervals (for instance, bi-monthly) for a maximum of three times until enough participants had been recruited, or the data collection period had ended. The resulting response rate of nearly 20% allowed us to reach our planned sample size.

### 2.3. Ethical Approval

The study was approved by the relevant ethical committees of both universities (registration number: 0058642/21 for Unimib and FHMS 20-21 157 EGA for UoS), and informed consent was reconfirmed verbally immediately before the interview and evidenced by digitally signing the consent form provided by the researcher.

### 2.4. Data Analyses

Interview data were thematically analysed using the framework approach [45,46,47]. The thematical analysis followed these steps: 1. familiarizing with the data; 2. generating initial codes; 3. constructing themes; 4. reviewing potential themes; and 5. defining and naming themes. The analysis took an exploratory, inductive approach to capture experiences on mental health of university students. One researcher (I.R.) first read each transcript independently, highlighting initial themes constructed from the data. Then, codes were organised into higher-order themes that represented relevant contents of students’ perspectives. Themes were reviewed by C.T. and J.A. separately, and differences in themes were discussed, leading to refinements to the original codes. A hierarchical thematic framework emerged as data analysis progressed. The final coding structure was discussed and agreed upon by all authors. Transcripts were recoded according to the finalised framework by I.R. Data were then extracted into matrices following a structured approach to facilitate identification of patterns within and between different groups of students (for instance, Unimib vs. UoS; male vs. female; living at home vs. living in university accommodation) [46,47]. The framework matrices were reviewed independently (by I.R., J.A., C.T.), and then through a panel discussion to build a consensus on emerging patterns.

## 3. Results

### 3.1. Sample Characteristics

We conducted a total of 33 individual interviews: 15 involving students from Italy and 18 with students from the UK, of which a total of 26 (79%) were females. The sample characteristics are reported in Table 1.

### 3.2. Themes and Sub-Themes

**Figure 1:**
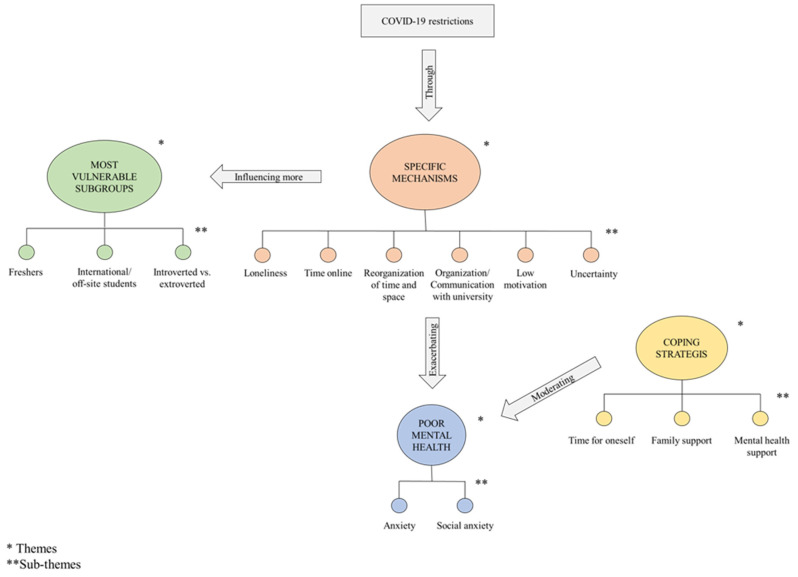
Explanatory model of the relationship between COVID-19 restrictions and poor mental health in students.

Four themes were identified from the analysis of the transcripts, including (i) anxiety exacerbated by COVID-19; (ii) mechanisms by which COVID-19 was related to poor mental health; (iii) the most vulnerable students; and (iv) coping strategies. Themes were further distributed across different sub-themes and ordered in the text according to their frequency across the transcripts (from most to least frequent). For a complete set of participants’ quotes please see Supplementary Table S1. The relationships within and between themes and sub-themes were explored leading to an explanatory model about how—and for whom—COVID-19 resulted in poor mental health in students based on interviewees’ perspectives. Indeed, COVID-19-related restrictions entailed specific circumstances, namely loneliness, more time spent online, reorganization of time and space, poor communication between students and the institution, low motivation, and uncertainty. These conditions acted as putative mechanisms of poor mental health in students during the pandemic period, mainly through the exacerbation of generalised and social anxiety. Most at risk subjects included freshers, students living far from their hometown, and students on the extremes of the introversion/extroversion spectrum, for whom restricted measures and loneliness were possibly more challenging. Finally, based on students’ accounts, anxiety symptoms were likely to be moderated by three different coping strategies: time for oneself, family and mental health support. The model is shown in Figure 1.

#### 3.2.1. Anxiety Exacerbated by COVID-19

Generalised and social anxiety symptoms were widely reported by interviewees in both countries, as the most prevalent negative effects of the pandemic among students.

- **Anxiety symptoms**

The majority of interviewees (67%) mentioned anxiety symptoms as a consequence of COVID-19. Students mainly described these as exacerbated worries about the capability of dealing with a wide range of academic issues, as well as concerns for the future.

*“One of the biggest effects on students’ mental health was anxiety that means a lot of things, for example anxiety about work and university stuff.” *

UoS, M.

*“Unfortunately, at this moment [during the pandemic] anxiety and concern for the future, for what will come next, in my opinion are very broad.” *

Unimib, F.

- **Social anxiety symptoms**

However, the perceived largest impact of the pandemic could be categorised as social anxiety symptoms. Especially considering the point of view of the youngest respondents, this condition was a likely consequence of mandatory social restrictions, finally resulting in the habit of self-isolation.

*“…[during the pandemic] a lot of people have been given the opportunity to lock themselves in their bedrooms and not have to talk to anyone, and they started to get used to this.” *

UoS, F.

*“I think it [pandemic] made people become more closed and more used to being online, which leads to you being more introverted.” *

UoS, M.

Some students reported becoming more introverted, increasing time spent online, and/or feeling anxious about re-integrating with others due to the possibility of being infected.

*“It [pandemic] can stress some people out quite a lot and make them anxious, as well as the risk of getting COVID or that family members could get COVID. So, they want to stay at home.” *

UoS, F.

A few students at the UoS described social anxiety symptoms as a progressive voluntary self-isolation, leading to being less comfortable in social contexts.

*“I think people have become comfortable staying at home and doing lectures on Teams. Now that they have to be in person, they feel a bit like forced to go back outside and this pushed them out of their comfort zone.” *

UoS, F.

Students from Italy also described this phenomenon, tending to characterize social anxiety symptoms as a challenging return to the pre-pandemic university life, namely to in person lectures and exams (outside their comfort zone).

*“…this fact of going to different lectures, the university in presence and this post-pandemic situation are very difficult. It is difficult to come back to reality.” *

Unimib, F.

*“…this anxiety to interact with students, with classmates, but also the lack of desire to attend lessons in person. Now that there is the choice on the lesson in person or remotely, many students, including me too, sometimes prefer to stay comfortable at home, I don’t know if it’s for a matter of social anxiety or comfort.” *

Unimib, F.

Few students referred to other mental health conditions associated with the pandemic, including sleep disorders, disordered eating and self-harm. Two students expressly reported an increase in depressive symptoms and only one described alcohol and substance abuse as a result of COVID-19.

#### 3.2.2. Mechanisms by Which COVID-19 Was Related to Poor Mental Health

Six different putative mechanisms were identified, with the most prevalent being loneliness and excessive time spent online.

- **Loneliness**

Twenty-two students, equally distributed between the two universities, identified loneliness as the most common mechanism involved in the relationship between the pandemic and poor mental health. All international students spoke about loneliness, though the sample was relatively small (*n* = 5). Isolation was intensified by social distancing measures and COVID-19 restrictions negatively impacted almost all participants, who described students as “*being in bubbles*”—bubbles was a term used by the UK Government to refer to size restricted social groups—and “*being stuck in their own rooms*”.

*“We were in bubbles. There was very limited social mingling.”*

UoS, F.

*“…thinking about this year of distance teaching… the worst thing was the fact of feeling alone caused by the circumstances that everyone had to be at home, not being able to attend the university in person, not being able to know their classmates and perhaps this also has generated precisely this sense of discomfort, that is feeling alone, a little abandoned.” *

Unimib, F.

Loneliness extensively affected university students because of the reported loss of usual social interactions with classmates and peers, with a higher impact on subjects living far from home who were requested to remain on campus, especially during the first lockdown.

*“You were just stuck in your room. And you couldn’t go home because the government had told you to stay where you are and that you can only travel for serious circumstances.” *

UoS, F [international student].

*“…some people who used to go out just for an hour or 20 min to talk to somebody suffered from the restrictions, especially during the main lockdown. Some students had to stay on the campus for the whole year without seeing their family. It has impacted everyone greatly.” *

UoS, F.

- **Time spent online**

Another consequence of the restrictions imposed by the pandemic was the increased amount of time spent on digital devices. Both UoS and Unimib students, especially the mature ones, expressed their views about the risks of “*always being online*”.

*“There were periods when there was only online, most of the campus was closed and therefore not the kind of the same sort of experience as a university student.” *

UoS, F.

The UK sample reported a related susceptibility to intentional self-isolation, leading to a sub-optimal experience of university life and a potential occurrence of social anxiety symptoms.

*“[One of the causes of poor mental health during COVID were] the lessons online ‘cause you were just on your own and most of our lessons are online. Some people don’t go out and I don’t go to the library: we have lectures in our rooms, on our computers.” *

UoS, F.

Similarly, students from Italy specified the role of excessive time online in the onset of mental health issues. Interestingly, students spoke about time spent online even related to study purposes, by disclosing a negative effect of an entirely online teaching mostly through an exacerbation of the distance between students and the educational institution (namely, the professor-students connection for academic success).

*“Even the contact with the lecturers, beyond the lectures that could be synchronous or asynchronous, was difficult. Then, in the end everything was done in front of the computer and in my opinion it was extremely alienating.” *

Unimib, F.

Indeed, lectures and exams held online were described as alienating and stressful, as well as favouring anxiety symptoms.

*“Online exams were really stressful, in particular the exams’ monitoring methods, however reliable they are. Many students were anxious or afraid to look at the screen for fear that the system will trigger the signaling for eye movements [Digital control system to detect exams cheaters].” *

Unimib, F.

- **Reorganisation of time and space**

COVID-19 restrictions and exclusive online learning frequently led to an unhealthy reorganization of individual time and space. This issue was raised equally at the two universities, by both female and male students. However, students from the UK mainly spoke about decreased opportunities to engage in social activities and the limited space to be shared with roommates for a longer time.

*“…you couldn’t go anywhere or do anything to take your mind off it. You didn’t have space for yourself.” *

UoS, F.

*“The pandemic has impacted students, because they had to stay with each other for such a longer period of time that they started to notice everyone defects. So, the pandemic ruined somebody’s relationships as well.” *

UoS, F.

*“There was a habit of staying there not doing anything. You wasted your time.” *

UoS, F.

Students at Unimib were more likely to report time and space-related issues caused by a lack of a strict division between working and spare time/space, resulting in an unbalanced predominance of time dedicated to their studies.

*“…it did not help that one began to depend only on the university, because that was to be done, university has never stopped, not even online. It really became an “ok I wake up, then I will follow this lesson, then this other one, then I will study for this exam, then I will go to sleep”. Time was just dictated by the university.” *

Unimib, F.

- Organization/communication with university

Failures in university organization and in communication with students during the pandemic were reported as a concern by the sample from Italy based, at least partly, on already existing communication issues. Students described feeling abandoned due to the lack of preparedness in terms of educational priorities with “*university left just as the last thing to think about*”.

*“…some internal communication difficulties, have come to light more clearly [during the pandemic].” *

Unimib, F.

*“University was left just as the last thing to think about, so the students felt a little neglected even with the fact that at the beginning of the pandemic, but also during the pandemic, you had no idea of how to take exams, find books, or get the information on the timetables. Many of those things happened for months. No one knew how to handle these things.” *

Unimib, M.

Interviewees reported a deep distance between students and lecturers following an inappropriate arrangement of online lectures and exams.

*“I missed going to the exam and seeing the lecturer, therefore breaking a barrier that was physical. With online exams and teaching, the distance between the figure of the teacher and you as a student persisted.” *

Unimib, F.

*“[One of the sources of poor mental health was] the distance between students and lecturers in the pandemic period. Almost a surreal situation.” *

Unimib, M.

- **Low motivation**

Low motivation was identified by five students from Italy as a consequence of the pandemic restrictions leading to poor mental health. In the most part, this was reported as a struggle to approach study, poor concentration, and withdrawal from studies.

*“There were many withdrawals from study attributable to demotivation [during the pandemic].” *

Unimib, F.

*“…perhaps also due to the COVID period, I personally felt a difficulty precisely in approaching studying, in concentrating, with low motivation, certainly linked, in my opinion, to university workload and also other things that I have had to face at the same time.” *

Unimib, F.

One of the students from the UK shared their opinion about low motivation, mostly referring to a decreased desire to devote time even to sport and social activities.

*“I do a couple of sports and you can tell that during COVID obviously we were unable to do that and now, even though we still show up to training, we are less motivated.” *

UoS, F.

- **Uncertainty**

During the pandemic, isolation and poor communication with the institutions fed the sense of uncertainty about the future and growth opportunities, both in Unimib and UoS students’ opinion.

*“During COVID-19 there wasn’t anything defined or certain, you know, circumstances were always changing.” *

UoS, F.

This was mainly experienced by those living at home with their parents, who referred to having a lower sense of independence and self-determination.

*“…uncertainty about the future was exacerbated by COVID.” *

Unimib, F.

*“During COVID, the perspective is always kind of much grayer than it was before. Will I be able to go abroad to have an experience? I do not know.” *

Unimib, F.

Moreover, sharing negative work experiences (for instance, job loss) with relatives might be considered as a possible moderator of the uncovered sense of uncertainty.

*“…some students, as well as their relatives, lost their jobs, and they felt a sense of uncertainty.” *

UoS, F.

#### 3.2.3. The Most Vulnerable Students

Freshers, as well as students living far from home, and subjects on the extremes of the introversion/extroversion spectrum were described as most at risk of poor mental health during the pandemic.

- **Freshers**

Loneliness and more time spent online decreased social opportunities, especially among first year students. They were considered to be the most vulnerable subgroup by several interviewed students (33%). Indeed, it was widely expressed by students in the UK and Italy that an essential part of the academic experience is the chance to meet new peers during the transition from secondary school to university.

*“A big part of university, especially in the first year, it is to meet people, and it was not possible during the pandemic* *.” *

UoS, F.

*“I was quite affected because the first year is when you make all your friends and get to know people and you couldn’t do that. It’s because it was first year that the pandemic made it like ten times worse.” *

UoS, F.

Two young students also spoke about the impact that COVID-19 had on their high school education, making them less ready for university.

*“Obviously COVID prevented freshers from going to school for so long and doing so many things. I think it could be quite a quick transition [from high school to university].” *

UoS, F.

*“…in particular, during COVID, they found themselves going from high school last year to university and were catapulted into the university world without knowing anything. They could have bad impression about the relationship with teachers.” *

Unimib, M.

Moreover, Unimib students highlighted for freshers a more challenging return to normality after the pandemic, including a poorer relationship with the institution.

*“Many freshers told me about the effort of having started the university path [course] alone and that even loneliness makes them very anxious, the fact of not being able to compare with anyone, they struggle so much with this.” *

Unimib, F.

*“Freshers are not used to the environment, and they may have some problems in starting in person lectures.” *

Unimib, F.

- **International/off-campus students**

Interviewees, mainly from the social degree programmes, identified students living far from home (international and off-campus) as a vulnerable population during the restrictive measures, given the likely sense of loneliness. This scenario was exacerbated by the feeling of being in a foreign environment.

*“I am really far from home, and it was a problem for me especially during COVID, because I felt very lonely, and I have to do a lot of things alone also at home. I had a lot of responsibility.” *

UoS, F. [international student].

*“…when we restarted to go out a little bit, I had a hard time making friends, because in any case everyone was in their own house. We never really found a moment to be together, to get to know each other better. Thus, in my opinion it was worse for off-site people because, in addition to being far from home, they didn’t even know the city they lived in.” *

Unimib, F.

Students from Italy also raised practical issues in terms of barriers during the pandemic when looking for medical support and trying to access health services.

*“…to be an off-site student during the pandemic was a bit problematic. I have discovered that in Lombardy to be able to book a specialist visit to the hospital you need the regional health card. Moreover, there have been some guys who have had problems in the sense that they were in contact with [COVID] positives, they called the healthcare system, but they didn’t get any information. Thus, during COVID it was really difficult for off-site students also from a practical point of view.” *

Unimib, F.

- **Introverted vs. extroverted students**

Since socialisation was described as an essential aspect of the university experience, opposite social skills, namely being an extremely extroverted or introverted individual, were seen as a possible source of vulnerability during the pandemic. According to some students, introverted people, who might take advantage of class time to socialise, were likely to be more vulnerable to pandemic restrictions as compared to their counterparts.

*“Some of the more outgoing people might have tried and find a way to socialize also during the pandemic, but maybe people who were relying on meeting people through lectures weren’t able to do that because everything was online.” *

UoS, F.

*“…especially the more introverted suffered during COVID because they tend to stay more closed.” *

Unimib, F.

Similarly, according to some interviewees, students at the other end of the spectrum (namely, extroverted people) were identified as a vulnerable target as well, since they were considered as less used to being alone.

*“The pandemic affected me in a way that wasn’t ideal. I don’t like being alone. I am a true extroverted person.” *

UoS, F.

*“The pandemic really took a turn on the mental health of people. Especially people that are a bit more extroverted and who like being outside and being with friends. They felt lonely because they couldn’t do what they did before.” *

UoS, F.

#### 3.2.4. Coping Strategies

Respondents described a variety of coping strategies that could help university students to overcome the negative effects of the pandemic.

- **Time for oneself**

Reorganization of time and space produced a greater amount of free time. Both UoS and Unimib students, especially women, recognised that taking advantage of leisure time helped in coping with the pandemic. Students from the UK suggested using time to explore new hobbies and interests, as well as to reunite with family, when possible in line with national directives, since campus life is generally distant from their hometown.

*“It [pandemic] gave me the time to sit down and reach out, ask for help and actually work on myself.” *

UoS, F.

*“…COVID had a negative impact for some of students, but not for me. When COVID started, I flew home, and I was home for almost seven months, and I really enjoyed it because being away from home was something I haven’t really got used to. And I had time to do a lot of things, activities for myself.” *

UoS, F.

For Unimib students, the pandemic meant additional time to devote to study since there are no distractions at all, thus benefiting students who also had a job.

*“I have conflicting ideas [about the effects of the pandemic on mental health]. Paradoxically, the first period, especially the first lockdown period, therefore March-April-May 2020, helped me a lot because, having no commitments that took away my time to study, I was able to attend courses and take exams. It was a very good semester from that point of view.” *

Unimib, F.

*“…on the other hand, among the benefits, there were for those working students, who still continued to carry out activities during the pandemic or even considered their opportunity to review online lessons in order to better understand them.” *

Unimib, F.

- **Family support**

Students, mostly from Italy and in particular those who lived far from home and reported a good relationship with relatives, specifically recognised family support as an effective strategy to cope with the pandemic challenges.

*“I was lucky, because anyway I was at home with my family during the first lockdown. I’ve never been alone, and it is important for mental health.” *

Unimib, M.

*“I was blessed to live in a peaceful environment with healthy relationships within the family. For example, from this point of view, I was lucky because I had the opportunity to go home. I returned home, I live in a very large house, we are five, I have a brother and a sister, so I didn’t go through the lockdown alone but with my family support.” *

Unimib, F.

- **Mental health support during the COVID-19 pandemic**

Mainly, students enrolled in scientific programmes considered crucial university strategies for mental health support, regardless that these were actually provided to cope with the impact of the pandemic. In particular, one interviewee spoke about helpful interventions delivered by the university in the form of informative webinars.

*“During COVID there was a series of events related both to how to carry on the career during the pandemic, and in general to mental health support. It was really useful.” *

Unimib, F.

Other students focused on unmet needs, stating that the university should have done more during this challenging period.

*“You were not in university all the time because of COVID, and you were isolated and therefore maybe the university would have needed to organize more discussions with students about the support of mental health and explain exactly what the support is.” *

UoS, M.

*“…to arrange some online session to meet with other people and speak about mental health. It could be useful especially in relation to COVID.” *

UoS, F.

### 3.3. Comparison between Unimib and UoS

Despite most of the sub-themes were shared by students from both universities, some differences emerged when comparing Italy and the UK.

At Unimib, students identified academic worries and the not entirely adequate organization of the university system as one of the main mechanisms by which COVID-19 was related to poor mental health. Conversely, UoS students described the impact of the pandemic on mental health as due to the loss of social connectedness, seen as an essential aspect of the academic experience. Moreover, students based in Italy, who usually share their accommodation with parents, highlighted the challenging management of individual space among other potential mechanisms leading to anxiety symptoms.

Finally, differences in terms of coping strategies as regards the pandemic were detected across the transcripts. Students from Italy identified family support and being able to dedicate more time to study as beneficial, while the UK sample devoted the greater amount of free time to personal growth.

## 4. Discussion

To our knowledge, this is the first cross-country qualitative study on the impact of COVID-19 on the mental health of university students. The findings provided a model for understanding how—and for whom—COVID-19 restrictions were likely to result in poor mental health. Specific factors, mainly loneliness and more time spent online, were identified as putative mechanisms underlying the relationship between the pandemic restrictions and mental health. Generalized and social anxiety symptoms were described as the main conditions reported by students, with freshers, students living far from home, as well as those who were more extroverted or introverted, being considered the most vulnerable subgroups. Our findings corroborate previous research that described an increased likelihood of reporting anxiety symptoms during the pandemic, mainly in higher education settings [18,19,20,21]. Indeed, academic and social challenges entailed by the restrictive measures imposed represent a possible source of psychological pressure [10,48,49]. In addition, in line with available evidence, loneliness and self-isolation seem specific triggers for social anxiety symptoms [50,51]. Consistently, increased time spent online was reported during the pandemic. This was perceived both as an opportunity to remain connected, and a potential risk factor for poor mental health [52,53]. 

On the one hand, virtual networks and communities on social media platforms were often deemed appropriate by higher-education students for self-disclosure or help-seeking possibly encompassing warnings on different stressors affecting psychological wellbeing during outbreaks [54,55]. On the other hand, spending more time online, including compulsory online-only learning, represented a reason for self-isolation and unhealthy management of individual time and space [16,25,26]. Although evidence supports the negative effect of excessive use of digital devices on the mental health of young people [56,57,58], findings on the impact of solely online learning are mixed [59,60,61,62,63].

Despite differences in the lockdown restrictions between Italy and the UK [64], students from both countries agreed about the impact of COVID-19 on feelings of uncertainty and poor motivation. Indeed, uncertainty about the future is a major stressor in young adults who are striving to find their roles both socially and in terms of education and work [65,66]. This was likely intensified by the pandemic, together with loneliness and excessive time online, with students experiencing deep low motivation in academic and social activities [22,23,24], probably more than expected in standard lifetime transition states [1,67], and affecting particularly first-year and international students [1,4,30]. Indeed, the first year of university can be a particularly lonely time for undergraduate students both in Italy and the UK [68,69,70]. Notably, university friendships are significant in favouring the adjustment to an unfamiliar social environment, thus representing a support for international or off/campus students [31,32]. A link between the opportunity to gain and carry out specific social skills and the likelihood of psychological symptoms was highlighted also in our study [71,72]. However, it is likely that the pandemic made the difference since, the role of introversion on social anxiety and loneliness disclosed by responders [73] suggest wide changes in social habits during the pandemic, likely risk factors for psychological distress also within the extroverted subgroup [74].

Despite most patterns were shared between the two countries, some differences emerged when comparing students from Italy and the UK. At Unimib, the impact of COVID-19 on mental health was explained as foremost resulting from academic worries and not the entirely adequate organization of the university system. Evidence showed that university students routinely experience academic pressure [75,76]. Exams are identified as the primary source of stress, followed by work overload, with a different impact related to some specific conditions, namely degree programmes and year of course [75,77]. Poor organization of learning plans, along with a distant relationship between students and lecturers during the pandemic were additionally highlighted by respondents from Italy. According to previous research, organisational problems, inadequate teaching supervision, and teacher-student conflictual interactions negatively impact on students’ psychological well-being [28,29]. Conversely, UoS students described the impact of the pandemic on mental health as due to the loss of social connectedness, seen as an essential aspect of the academic experience [70,78]. Indeed, lifestyle factors, namely the different, typical living accommodation, might have played a key role in the perceived impact of the pandemic on students’ mental health [79]. In the UK, living on campuses or in shared residences is the most popular choice of accommodation for university applicants [4,80]. Therefore, several challenges are associated with moving from home into student accommodation, such as living with strangers, developing independence, and managing domestic duties and needs [40,81]. Nevertheless, also our findings show campus life more as an opportunity to enrich their social experiences, thus complaining about the loss of social connectedness during the pandemic [70,78]. Moreover, students based in Italy, except international and non-resident students, usually live with parents [82,83], thus experiencing both pros and cons of their living arrangements. During the COVID-19 outbreaks, the challenging management of individual space was a potential disadvantage highlighted by Italian students sharing their accommodation with relatives [84,85].

Finally, strategies for coping with the pandemic from the interviews might have been similarly influenced by the specificity of the cultural environment. Students from Italy, mostly living with their parents, identified family support and being able to dedicate more time to study as beneficial, while the UK sample devoted the greater amount of free time to personal growth. Both strategies, family support, and time for oneself, have previously been identified as adaptive behaviours in response to stressful events [86,87,88,89]. Mental health support to be provided by the educational institution was likewise represented across the two universities as a relevant coping strategy. Indeed, during the pandemic, mental health interventions tailored on students’ specific needs showed effectiveness [34,90].

### Limitations

Our findings should be interpreted with caution considering some methodological limitations. First, although we attempted to diversify the sample to include various groups of students in terms of sociodemographic characteristics, male students were under-represented, thus preventing a proper gender-based comparison. Despite this, there was an appropriate distribution across age, degree programmes and living conditions, interviewees might be students who were already well engaged, to some extent outgoing, and interested in mental health topics. Although generalizability is not an issue inherent to qualitative research, this scenario may suggest an additional potential selection bias with overestimation/underestimation of the condition of interest. Second, the principal investigator’s education and background might have influenced the interview contents and subsequent interpretation, through a possible restricted focus on mental health issues. Nevertheless, interview questions were research-based, and piloted before the start of the study to ensure understandability and face validity in relation to topics covered, as well as maximum relevance.

Finally, due to some difficulties encountered in online recruitment, the interviews were conducted over a relatively long interval of time (from September 2021 to April 2022), thus potentially undermining students’ feelings and perspective about the pandemic issues also in terms of recall bias about past exposure or outcomes. However, the interviews were held in a private setting, which has been shown to minimise worries about privacy and anonymity, thus allowing participants to freely express themselves and take their time to explain their perspective [91].

## 5. Conclusions

The current study highlights the likely psychological burden among university students during the pandemic and the key role of mental health support in university settings. Our findings add to the existing evidence supporting the need for measures to improve communication within educational institutions, as well as to encourage social connectedness, as one of the elements of university experience. The complex relationship between stressful events, such as a pandemic, and poor mental health, advocates interventions aimed at strengthening positive coping strategies, especially targeting vulnerable students. Further research is needed to provide additional insight into this field, taking into account also national cultural specificities.

## Figures and Tables

**Table 1:** Sample Characteristics.

Characteristics	Total	Italy	UK
	N = 33 N (%) or Median (IQR) *	N = 15 (45%) N (%) or Median (IQR) *	N = 18 (55%) N (%) or Median (IQR) *
***Sex***			
Women	26 (79%)	12 (80%)	14 (78%)
Men	7 (21%)	3 (20%)	4 (22%)
***Age*** *, years.* Median (IQR) *	22 (21–22.5)	22 (21–23)	21 (20–25)
***Nationality***			
Italian	15 (45%)	15 (100%)	0
British	13 (40%)	0	13 (72%)
Other	5 (15%)	0	5 (28%)
***Year of study***			
First	7 (21%)	2 (13%)	5 (28%)
Second	12 (37%)	4 (27%)	8 (44%)
Third	5 (15%)	2 (13%)	3 (17%)
Fourth	6 (18%)	4 (27%)	2 (11%)
Fifth-Sixth	3 (9%)	3 (20%)	0
***Degree Programme***			
Applied/Formal Sciences ^1^	4 (12%)	2 (13%)	3 (17%)
Economic/Legal Sciences ^2^	6 (18%)	4 (27%)	2 (11%)
Medical Sciences ^3^	9 (27%)	3 (20%)	6 (33%)
Natural Sciences ^4^	6 (18%)	1 (7%)	4 (22%)
Psychosocial Sciences ^5^	8 (24%)	5 (33%)	3 (17%)
***Accomodation***			
On Campus	8 (24%)	1 (7%)	7 (39%)
With family	11 (34%)	6 (40%)	5 (28%)
Alone	5 (15%)	3 (20%)	2 (11%)
Accommodation with roommates in university town	9 (27%)	5 (33%)	4 (22%)

* IQR: Interquartile range. ^1^ Applied/Formal sciences: biomedical engineering; computer sciences; maths. ^2^ Economic/Legal sciences: economics; hospitality and tourism; law; politics. ^3^ Medical sciences: medicine and surgery; nursing; nutrition; paramedics, veterinary medicine. ^4^ Natural sciences: biochemistry; biology; biomedical sciences; geology. ^5^ Psychosocial sciences: education sciences; intercultual communication; psychology; sociology.

## Data Availability

The data presented in this study are available in Supplementary Materials and can be downloaded at: https://www.mdpi.com/article/10.3390/ijerph20054071/s1.

## Supplementary Materials

The following supporting information can be downloaded at: https://www.mdpi.com/article/10.3390/ijerph20054071/s1, File S1: Topic guide for interviews; Table S1: Complete set of participants’ quotes divided by themes and sub-themes.
