# Supplementary material for: Mental Health and COVID-19 in University Students: Findings from a Qualitative, Comparative Study in Italy and the UK
Source: Int J Environ Res Public Health. 2023 Feb 24;20(5):4071. doi: 10.3390/ijerph20054071 (PMC10001873; doi:10.3390/ijerph20054071)
Supplement: Supplementary file 1 [file ijerph-20-04071-s001.zip › ijerph-2146504-supplementary.pdf]

UNIVERSITA' DEGLI STUDI DI MILANO BICOCCA

UNIVERSITY OF SURREY

Supplementary File S1. Topic guide for interviews

Caring and **A**ssessing **M**ental health of student **P**opulations at **U**nimib and uni**S**urrey**: the CAMPUS study**

Introduction

Hello, my name is Ilaria, I am a PhD student at the University of Surrey, and I would like to invite you to participate in this research project, which forms part of my PhD research.

The purpose of this study is: to assess and compare mental health among students from UniSurrey and the University of Milano Bicocca (UNIMIB, Italy), and to explore associated factors using a survey design; to assess mental health and well-being among UniSurrey and UNIMIB students during the COVID-19 outbreaks; to identify the areas of greatest interests and the main critical issues about students' psychological wellbeing for the design and evaluation of future preventive digital interventions.

This present stage of the study will involve interviewing university students about their perspectives and experiences of mental health and psychological well-being. The conceptual framework was developed starting from the existing literature on the field.

These interviews will help develop an online survey aimed at investigating different domains of mental health in university students.

In-depth interviews will be conducted by the principal investigator through an online platform (MS Teams) and will be recorded.

We will collect information on students' awareness and knowledge about university students' mental health and all the services offered to improve their well-being. Each interview will follow a semi-structured topic guide (see below), with no right or wrong answers, but in the form of a conversation.

Interviews will last between 20 to 30 minutes. Informed consent will be audio-recorded and checked verbally immediately prior to commencing the interview.

Participants will be asked for consent to have the interview audio and video recorded. Interviews will be transcribed for substantive content by the researcher, and they will be destroy once transcribed.

Participants will be informed they are free to discontinue an interview at any time until it concludes and may withdraw the data from that interview for up to seven calendar days following that interview by informing the researcher, without needing to give a reason.

Your personal data will be kept securely in accordance with data protection guidelines, and only be accessible to the immediate research team or responsible persons at the University.

Please could you confirm for me if you agree to participate in this study? Please could you confirm that you agree with the following points:

[read list from consent form]

Are you happy for me to record our interview? [If yes, start recording]

Opening questions

- Which degree programme are you enrolled in?
- What year are you in?
- How old are you?
- What is your nationality?
- Are you an international student?
- Can I ask you something about your accommodation?

University students and mental health / psychological well-being.

- Do you think it is important to talk about mental health in university students?
- Before asking you more specific questions, can I start by asking how many people you think suffer from mental health problems at university? Are there specific subgroups of students that could be more at risk of mental health issues?
- In your opinion, what could be the causes of a low level of psychological well-being? Do you think it could be connected with academic worries, loneliness, change in habits, or other reasons?

University students and mental health's specific domains

- What do you think are the most common psychological/mental health problems faced by university students among depression, anxiety, stress, substance abuse, self-harm, suicidal ideation, eating disorders, poor sleep quality?
- Are there any other specific mental health problems you consider common among university students?
- In your opinion, what are the most common manifestations of a low level of psychological well-being?

Mental Health and Covid-19

- How do you think Covid-19 has influenced the students' psychological wellbeing?
- In your opinion, what are the main psychological problems related to the pandemic?

Intervention

- PREVENTION PROGRAMME and CENTERS FOR WELLBEING

- In your opinion how often do university students seek help to improve their psychological difficulties?
- What do you think can be done to improve students' mental health? In what ways could UniSurrey service for students' mental health be improved?
- What do you know about prevention programmes offered by UniSurrey to improve students' mental health?
- Are there any personal barriers, such as reluctance to seek help or stigma, which delay students accessing services?
- Are there any service-related barriers, such as overbooking and limited staff, which delay students access to the service?

<u>Areas to explore:</u>

- Referral route
- Waiting times
- Information sharing

- DIGITAL

- What do you know about digital interventions for the identification or treatment of psychological difficulties?
- Could a digital intervention be useful to prevent and treat low levels of psychological well-being in university students?

- Have you ever used one of these digital interventions (app, website)? If so, how did you find it?
- Which type of contents is it useful to include?

Concluding questions

- Are there any questions that we have not asked you that you were expecting? Why do you think this is important to ask about?
- If I have any further questions following on from today, would you mind if I contacted you by telephone or email to clarify?
- Would you like a copy of the final report when the study finishes? If so, how should we send this to you? (Get email/postal address and give them indication of when this might be).

Thank you very much for your time today.

Supplementary Table S1. Complete set of participants' quotes divided by themes and sub-themes.

Themes and Sub-themes	Quotes
**ANXIETY EXACERBATED BY COVID-19***	
**Anxiety symptoms****	• "Some students may be quite anxious to do things with COVID." UoS, F<br>• "With the pandemic maybe you can develop a new mental health disorder, like anxiety or depression." UoS, M<br>• " One of the biggest effects on students' mental health was anxiety that means a lot of things, for example anxiety about work and university stuff." UoS, M<br>• "Unfortunately, at this moment [during the pandemic] anxiety and concern for the future, for what will come next, in my opinion is very broad." Unimib, F<br>• " [During the pandemic] loneliness makes students very anxious." Unimib, F<br>• "…stress and anxiety exacerbated by COVID, with the desire to control everything." Unimib, F
**Social Anxiety****	• "Maybe social anxiety has increased due to the pandemic." UoS, F<br>• "…[during the pandemic] a lot of people have been given the opportunity to lock themselves in their bedrooms and not have to talk to anyone, and they started to get used to this." UoS, F<br>• "I think people have become comfortable staying at home and doing lectures on Teams. Now that they have to be in person they feel a bit like forced to go back outside and this pushed them out of their comfort zone." UoS, F<br>• "I think it [pandemic] made people become more closed and more used to being online, which leads to you being more introverted." UoS, M<br>• "It happened to me that lecturers asked to keep the cameras on but then it was like being there still alone, then the desire passes, turn off, at least you can stay there in your pyjamas, in shorts, in your comfort, alone." Unimib, F<br>• "It [pandemic] aggravates a little social anxiety." Unimib, F<br>• "For me, COVID was an occasion in which, let's say, problems related to social anxiety arose in my opinion." Unimib, F<br>• "It may be a bit harder to get out if things are online and now the pandemics is getting better and there are more opportunities to go out. But since everything is online and it's harder to make friends as well and talk to people and to find the desire to go out." UoS, F<br>• "…this fact of going to different lectures, the university in the presence and this post-pandemic situation are very difficult. It is difficult to come back to reality." Unimib, F<br>• "One thing that really frightened me was the fact that many students preferred the remote exam precisely because they were in their home environment. In September we should return with the exams in the presence. But in my opinion it will be a very difficult start." Unimib, F<br>• "…this anxiety to interact with students, with classmates, but also the lack of desire to attend lessons in person. Now that there is the choice on the lesson in presence or remotely, many students, including me too, sometimes prefer to stay comfortable at home, I don't know if for a matter of social anxiety or comfort." Unimib, F<br>• "…a subsequent work to be done that many people, even with myself,

	are still in the process of evolution, that is, to balance the situation again, to find a balance both with us and precisely by returning to first with all the others. It is necessary as a coping strategy." Unimib, F<br>• "It [pandemic] can stress some people out quite a lot and make them anxious, as well as the risk of getting COVID or that family members could get COVID." UoS, F<br>• "…then you're exposed to COVID and then you're worrying you're going to bring it home to your family." UoS, F<br>• "Students could be worried about getting out and go to visit their family members. Some people may not go out to big events where there are loads of people 'cause they don't want to get COVID." UoS, F<br>• "I was so anxious of getting COVID, so I always stayed at home." UoS, M
**MECHANISMS BY WHICH COVID-19 WAS RELATED TO POOR MENTAL HEALTH***	
**Loneliness****	• "Because we were in lockdown students were alone, they didn't have anything to do, it's clear to me that COVID impacted mental health a lot, in general, like not only students but especially students." UoS, F<br>• "…you don't realize how good it is to socialize and meet people on a daily basis until you're not able to do that anymore." UoS, F<br>• "We were in bubbles. There was very limited social mingling." UoS, F<br>• "…but especially the starting last June was very isolated." UoS, M<br>• "They [students] became isolated, some of them couldn't see their family or their friends or their loved ones, which can have a really big impact." UoS, F<br>• "I don't think it [pandemic] helped up like it definitely increased loneliness." UoS, F<br>• "[One of the main causes of poor mental health] was loneliness because a lot of people haven't had that physical interaction for quite some time." UoS, F<br>• "I think it was probably quite profound for my year because we were in bubbles." UoS, F<br>• "You were just stuck in your room. And you couldn't go home because the government had told you to stay where you are and that you can only travel for serious circumstances." UoS, F<br>• "Even lessons are online, and you can feel quite isolated." UoS, F<br>• "…under lockdown, people have felt loneliness and social isolation and it has had a big impact on mental health and on the mood." UoS, F<br>• "…some people who used to go out just for an hour or 20 minutes to talk to somebody suffered from the restrictions, especially during the lockdown. Some students had to stay on the campus for the whole year without seeing their family. It has impacted everyone greatly." UoS, F<br>• "…the social isolation and all the loneliness. The fact that people haven't seen each other face to face for a large amount of time increased the misunderstandings between people." UoS, M<br>• "…surely the fact of not being with your classmates all day, I don't know, from home it's you and yourself, the social part is missing, even the two words before the exam to dampen anxiety. Having classmates next to class is a bit of a certainty. I think this is the most negative thing." Unimib, F<br>• "[One of the main causes of PMH during COVID was] loneliness, lack of contact with others." Unimib, F<br>• "…especially in the first semester that we were in the red zone, it seems to me, or something like that, human contact was almost non-existent."

	Unimib, F<br>• " During COVID, we didn't have internships, we couldn't play sports, we couldn't go out." Unimib, M<br>• "The Government said: <<From today onwards you will remain closed at home contacts with no one>>. I know people who made the first lockdown totally by themselves at home." Unimib, F<br>• "In other words, in my opinion it [COVID] has played above all in those who are perhaps used to studying at the university, so they could not go out and they have lived it more." Unimib, F<br>• "…the fact of being alone, in front of a computer, perhaps with all the cameras off and the teacher speaking, you almost miss the people who make a mess at the back of the class, so yes it was not pleasant." Unimib, F<br>• "…thinking about this year of distance teaching… the worst thing was the fact of feeling alone caused by the circumstances that everyone had to be at home, so not being able to attend the university in presence, not being able to know their classmates and perhaps this also has generated precisely this type of discomfort, that is to feel alone, a little abandoned." Unimib, F<br>• "…distance between students. They were detached and isolated from the world." Unimib, M
**Time spent online****	• "…especially because of stuff being a lot online, people have been very closed in." UoS, F<br>• "There were periods when there was only online, most of the campus was closed and therefore not the kind of the same sort of experience as a university student." UoS, F<br>• "Students would just be with lectures online and nothing else." UoS, M<br>• "I think a lot of students don't like online learning, and it caused problems with mental health." UoS, F<br>• "…you can't even leave from home for lectures 'cause they were online. Everything was online, and it was not good for mental health." UoS, F<br>• "[One of the causes of poor mental health during COVID were] the lessons online 'cause you were just on your own and most of our lessons are online. Some people don't go out and I don't go to the library: we have lectures in our rooms, on our computers." UoS, F<br>• "I think it's made people become more closed and more used to being online, and it is not good for mental health." UoS, M<br>• "The online lectures increase the anxiety." UoS, F<br>• "Online exams were really stressful, in particular the exams' monitoring methods, however reliable they are. Many students were anxious or afraid to look at the screen for fear that the system will trigger the signaling for eye movements [Digital control system to detect exams cheaters]." Unimib, F<br>• "…doing the lessons at a distance, online was a source of low mental health." Unimib, F<br>• "Even the contact with the lecturers, beyond the lectures that could be synchronous or asynchronous, was difficult. Then, in the end everything was done in front of the computer and in my opinion it was extremely alienating." Unimib, F<br>• "…certainly, the remote situation did not even help mental health." Unimib, F<br>• "I remember that in the first semester of COVID, then the second semester of last year, yes, I spent the whole day online at the computer

	following the lessons and it was not good for my mental health." Unimib, F
**Reorganisation of space and time****	• "…You couldn't go anywhere or do anything to take your mind off it. You did't have space for yourself." UoS, F<br>• "The pandemic has impacted students, because they had to stay with each other for such a longer period of time that they started to notice everyone defects. So, the pandemic ruined somebody's relationships as well." UoS, F<br>• "There was a habit of staying there not doing anything. You wasted your time." UoS, F<br>• "…when you have a meeting online you lose all the part regarding the spare time, so going to drink a coffee or a beer all together. The management of time was bad." UoS, M<br>• "…the problem was this little differentiation of space and time of the places in which one lives and studies." Unimib, F<br>• "The overload of things to do at the university added to the things at home, with no separation of time and space." Unimib, F<br>• "…even being banally in the same room, like I live in a two-room apartment that I have to stay in the same four walls every day, all day in front of a computer, passing from synchronous lessons to asynchronous lessons to study on the computer." Unimib, F<br>• "…it did not help that one began to depend only on the university, because that was to be done, university has never stopped, not even online. It really became an "ok I wake up, then I will follow this lesson, then this other one, then I will study for this exam, then I will go to sleep". Time was just dictated by the university." Unimib, F<br>• "More or less everyone found themselves in difficulty with the exams and the fact that many have not been able to pass them and then overlap over time, this is seen not in June, not in September but in this period here and then January and this session here in June and people find themselves with many more exams to give because last year they found themselves in trouble." Unimib, M<br>• "The only thing: the atmosphere of the last year was a bit alienating, in the sense that you get to the last one you finish the year without really realizing it, because it is not that there is an actual break from the end of lessons to graduation then it is just a bit of everything, a continuity. You have to organize yourself and don't have to get lost at the same time." Unimib, F
**Organization/communication with university****	• "COVID changes for the worse how everything operates in university." UoS, F<br>• "…some internal communication difficulties, have come to light more clearly [during the pandemic]." Unimib, F<br>• "…The contact with the professors, beyond the lectures that could be synchronous or asynchronous, during the pandemic became worse." Unimib, F<br>• "…let's say also in the management of the lessons themselves, precisely of the exams, that was as if, at least in the management of teaching, in my opinion it was a bit as if nothing had happened." Unimib, F<br>• "University was left just as the last thing to think about, so the students felt a little neglected even with the fact that at the beginning of the pandemic, but also during the pandemic you had no idea of how to take exams, find books, or get the information on the timetables. Many of those things happened for months. No one knew how to handle these

	things." Unimib, M<br>• "I missed going to the exam and seeing the lecturer, therefore breaking a barrier that was physical. With online exams and teaching, the distance between the figure of the teacher and you as a student persisted." Unimib, F<br>• "[One of the sources of poor mental health was] the distance between students and lecturers in the pandemic period. Almost a surreal situation." Unimib, M
**Low motivation****	• "I do a couple of sports and you can tell that during COVID obviously we were unable to do that and now, even though we still show up to training, we are less motivated." UoS, F<br>• "It [pandemic] caused lower performance due to lower motivation." Unimib, F<br>• "…there was a lot of demotivation due to COVID in university and in study." Unimib, F<br>• "…during the pandemic there was demotivation in starting a new path. Demotivation even for those who already had an activated path." Unimib, F<br>• "There were many withdrawals from study attributable to demotivation [during the pandemic]." Unimib, F<br>• "…perhaps also due to the COVID period, I personally felt a difficulty precisely in approaching studying, in concentrating, with low motivation, certainly linked, in my opinion, to university workload and also other things that I have had to face at the same time." Unimib, F
**Uncertainty****	• "During COVID-19 there wasn't anything defined or certain, you know, circumstances were always changing." UoS, F<br>• "…some students, as well as their relatives, lost their jobs, and they felt a sense of uncertainty." UoS, F<br>• "…uncertainty about the future exacerbated by COVID." Unimib, F<br>• "During COVID, the perspective is always kind of much grayer than it was before. Will I be able to go abroad to have an experience? I do not know." Unimib, F
**THE MOST VULNERABLE STUDENTS***	
**Freshers****	• "A big part of university, especially in the first year, it is to meet people, and it was not possible during the pandemic." UoS, F<br>• "Obviously COVID prevented freshers from going to school for so long and doing so many things. I think it could be quite a quick transition [from high school to university]." UoS, F.<br>• "I think people who started during a pandemic would have struggled to find friends or find friends quickly." UoS, F<br>• "I guess you know when you initially go to university, in the first year, and you find who to deal with. We were put in a bubble for learning, and you had to stay in your rooms, and you didn't mix with other people." UoS, F<br>• "I was quite affected because the first year is when you make all your friends and get to know people and you couldn't do that. It's because it was first year that the pandemic made it like ten times worse." UoS, F.<br>• "…when COVID started, you know, it was so hard for me to even find friends because I was on my 1st year." UoS, F<br>• "I can't imagine, especially maybe the new first year guys who don't know anyone and found themselves in these WhatsApp chats without knowing their mates." Unimib, F<br>• "Many freshers told me about the effort of having started the university

	path [course] alone and that even loneliness makes them very anxious, the fact of not being able to compare with anyone, they struggle so much with this.” Unimib, F.<br>• “Freshers are not used to the environment, and they may have some problems in starting in person lectures.” Unimib, F<br>• “…especially with the younger ones, everything was totally wrong during the pandemic.” Unimib, F<br>• “…in particular, during COVID, they found themselves going from high school last year to university and were catapulted into the university world without knowing anything. They could have bad impression about the relationship with teachers.” Unimib, M
**International/off-campus students****	• “I am really far from home, and it was a problem for me especially during COVID, because I felt very lonely, and I have to do a lot of things alone also at home. I had a lot of responsibility.” UoS, F<br>• “…when we restarted to go out a little bit, I had a hard time making friends, because in any case everyone was in their own house. We never really found a moment to be together, to get to know each other better. Thus, in my opinion it was worse for off-site people because, in addition to being far from home, they didn’t even know the city they lived in.” Unimib, F<br>• “…to be an off-site student during the pandemic was a bit problematic. I have discovered that in Lombardy to be able to book a specialist visit to the hospital you need the regional health card. Moreover, there have been some guys who have had problems in the sense that they were in contact with [COVID] positives, they called the healthcare system, but they didn’t get any information. Thus, during COVID it was really difficult for off-site students also from a practical point of view.” Unimib, F
**Introverted vs. extroverted students****	• “Some of the more outgoing people might have tried and find a way to socialize also during the pandemic, but maybe people who were relying on meeting people through lectures weren't able to do that because everything was online.” UoS, F<br>• “The pandemic affected me in a way that wasn’t ideal. I don't like being alone. I am a true extroverted person.” UoS, F<br>• “The pandemic really took a turn on the mental health of people. Especially people that are a bit more extroverted and who like being outside and being with friends. They felt lonely because they couldn’t do what they did before.” UoS, F<br>• “…especially the more introverted suffered during COVID because they tend to stay more closed.” Unimib, F
**COPING STRATEGIES***	
**Time for oneself****	• “It [pandemic] gave me the time to sit down and reach out, ask for help and actually work on myself.” UoS, F<br>• “…COVID had a negative impact for some of students, but not for me. When COVID started, I flew home, and I was home for almost seven months, and I really enjoyed it because being away from home was something I haven’t really got used to. And I had time to do a lot of things, activities for myself.” UoS, F.<br>• “I love to be with other people, but also at the same time the pandemic has helped me to understand the importance of having a personal hobby. I had the time to listen to a lot of podcasts and it helped me. in some way it was so much easier to connect to my friends because I

	know that they were all also locked up. And I had a lot of online contact with my family and my friends" UoS, F<br>• "I have conflicting ideas [about the effects of the pandemic on mental health]. Paradoxically, the first period, especially the first lockdown period, therefore March-April-May 2020, helped me a lot because, having no commitments that took away my time to study, I was able to attend courses and take exams. It was a very good semester from that point of view." Unimib, F<br>• "…on the other hand, among the benefits, there were those for working students, for whom still continued to carry out activities during the pandemic or even considering their opportunity to review online lessons in order to better understand them." Unimib, F
**Family support****	• "I was lucky, because anyway I was at home with my family during the first lockdown. I've never been alone, and it is important for mental health." Unimib, M.<br>• "I was blessed to live in a peaceful environment with healthy relationships within the family. For example, from this point of view, I was lucky because I had the opportunity to go home. I returned home, I live in a very large house, we are five, I have a brother and a sister, so I didn't go through the lockdown alone but with my family support." Unimib, F.
**Mental health support during the COVID-19 pandemic****	• "You were not in university all the time because of COVID, and you were isolated and therefore maybe the university would have needed to organize more discussions with students about the support of mental health and explain exactly what the support is." UoS, M<br>• "…to arrange some online session to meet with other people and speak about mental health. It could be useful especially in relation to COVID." UoS, F<br>• "During COVID there was a series of events related both to how to carry on the career during the pandemic, and in general to mental health support. It was really useful." Unimib, F

*Themes

**Sub-themes
